# From Deathbed to Database Using ICD10h: A Framework for Coding and Classifying Individual English Language Cause of Death Data Consistently Over Time, as Applied to Scotland 1855–1973

**DOI:** 10.1093/shm/hkaf077

**Published:** 2026-02-05

**Authors:** Alice Reid, Eilidh Garrett, Hannaliis Jaadla, Chris Dibben, Lee Williamson, Richard Tobin

**Keywords:** mortality, cause of death, mortality categorization, death registration, Scotland

## Abstract

This paper describes the development of, and principles behind, ICD10h, a system for the coding and categorisation of individual-level causes of death from 1855 to the present day. ICD10h provides an adaptation of the WHO's ICD10 (2016 version) which, by supplying additional codes for archaic terms together with a suggested categorisation, allows broad disease groups to be followed over time. It is a flexible system, which allows researchers to code, classify and study historic (h), individual level, and cause of death (COD) information in the English language, both in fine detail and at more aggregated levels. Our paper outlines the background and development of the system, before describing its principles and key characteristics. It demonstrates the system in action using individual level COD data for Scotland 1855–1973, as well as several smaller datasets and the published returns from the Registrar-General of Scotland.

## Introduction

The most influential theories about how and why mortality in Europe started to fall during the late nineteenth century rely on statistical series of causes of death. For example, both Omran's epidemiologic transition (describing the change from the dominance of infectious diseases primarily among the young to noncommunicable diseases concentrated in older ages) and McKeown's argument that it was improving standards of living which led to this change used death rates by broad cause of death (COD) categories published by the Registrar-General of England and Wales.^1^ The relatively recent, and increasing, availability of individual level death records that contain causes of death are revealing the instability of the apparently solid published categories which underpin these big-picture theories. Factors such as changing medical knowledge, the accessibility of doctors, registration systems, and changes in acceptable causes of death—issues that have always been known to affect particular causes of death—are prompting renewed questions about whether we can interpret the Registrar-General's statistics at face value.^2^ In other words, do differences in terminology reflect real differences and changes in character and prevalence of disease, or simply changes in fashion and medical knowledge?

This question is highly relevant to both medical historians and historical demographers, but previous authors have drawn attention to fundamental differences in the way that the two fields approach and interpret causes of death. Kunitz suggested that medical and population historians argue from different premises: medical historians are attuned to the particular and the local, giving them an excellent understanding of issues that prevent comparability between particular diagnoses and classificatory schemes over time, whereas historical demographers aim for generalisability, and use imperfect data in ingenious ways to shed light on patterns of disease in the past.^3^ This is reflected in different approaches towards COD categorisations: one focussing on individual causes where ambiguities can be specifically addressed, and the other favouring broad categorisations which make it more possible to study change over time.^4^ The different premises make mutual understanding and collaboration difficult to achieve, but Kunitz suggests that progress can be made if historical demographers do more to understand the particular ways that causes of disease and death were understood in the past, and if medical historians ‘recognize that some kinds of knowledge may indeed be generalizable, even though the data are highly particular’.^5^ Risse goes further, suggesting that both the particularity of insights from medical historians and the statistical nature of findings from demographic historians reveal the inherent instability of the knowledge of the past, and arguing that the combination of these disciplines can together create ‘a sturdier mosaic of the past’.^6^

This paper reports on a project that aims to make this dialogue easier: a historic COD coding and categorisation system, ICD10h,^7^ for use with large databases containing individual-level causes of death, that facilitates analysis at the levels of both specific disease or condition and wider groupings.^8^ The former can be used for detailed analysis of particular terms in specific contexts, or comparisons across context or times, while the latter can be used for broader statistical analysis. More importantly, the system is designed so the two levels can inform each other. In particular, as new knowledge about the use of particular disease terms is discovered, this can be incorporated into new or amended groupings for broader brush analysis. In this way it responds to a call made by Alter and Carmichael in 1996 for attention to the terms that describe causes of death, including ill-defined terms, vague causes, and proportions allocated to a ‘cause unknown’ category, and also for a coding system that does not simply try to fit older causes into the modern ICD system. Although the codes in our system are based on ICD10, the categorisations are more strongly based on nineteenth-century nosologies.

The aim of this paper is to introduce the coding system and to explain how it works for those wishing to use it, but we also illustrate different ways the codes and categorisations can shed light on both the general and the particular, with the help of examples using Scottish data. These examples compare broad COD categories with published data to assess the performance of the coding process, and a more focused example allows insight into the way that nineteenth-century clerks dealt with multiple causes on death certificates. Further examples illustrate the way that the different terms contributing to two broader causal groups (tuberculosis and diarrhoea) changed over time and varied between places.

The rest of our paper is organised as follows: first, we describe some of the issues involved in comparing causes of death over time and space, with particular reference to the Scottish context. We then introduce previous approaches to the treatment of historic causes of death and a justification of our approach. After that, we move on to an overview of ICD10h; the construction of ICD10h codes and some examples of the coding system at work; and the construction of a new classification for long-term comparisons. We then describe the application of the system to the Scottish dataset it was designed for, and finally, we illustrate the system using that data.

## Issues involved in comparing causes of death over time and space, with particular reference to the Scottish context

The sensitive interpretation of causes of death—whether at the level of an individual cause on a death certificate or the numbers of deaths attributed to a category in an official report over time—needs an appreciation of the social, economic, and geographical context, including the customary and legal circumstances surrounding the recording of death. Relevant factors include differences in the terminology used by doctors and lay informants, and the way that this interacted with differences in medical provision and attendance to produce geographical and social patterns in causes of death. Changes in medical knowledge and fashion affected the phrasing and complexity of the causes chosen by doctors and also the way that these were grouped and interpreted by statisticians. We start by describing the reasons that deaths from the same disease or condition might have been assigned different causes in the death register, before going on to consider the influences on the different ways these were abstracted into categories in an official report. Given the fact that ICD10h was designed to code and categorise Scottish deaths over a long time span, we pay particular attention to the Scottish registration context and the way that it developed, but also make comparisons with other contexts. These issues lead, at the end of the section, to a statement of the need for the sort of flexible coding and classification system that this paper describes.

Previous analysis has shown that terms for causes of death written on death certificates differed greatly depending on whether they were offered by a doctor, by a semi-trained informant such as a priest, or by a lay informant. Reid and Garrett showed that lay informants in Scotland would offer colloquial terms such as consumption, croup, childbirth, or old age, and quite often did not venture a cause at all. In contrast, doctors almost always offered a medical COD and used more scientific terms (e.g., phthisis or respiratory tuberculosis, bronchitis, puerperal eclampsia, or cardiovascular disease).^9^

It then becomes pertinent to ask why some causes of death were certified by doctors and others were not, and how this changed over time. Registration systems varied according to whether they required doctors to certify causes of death, or indeed, whether they would accept a cause offered by a non-medical informant. In some nineteenth-century settings, such as Copenhagen, it was mandatory for a dead body to be examined by a physician—both to ensure the patient was actually dead and to indicate the COD.^10^ Full coverage was obtained by subsidies that enabled the poor to comply, although this was not attempted in rural areas where there were far fewer physicians.^11^ In Scotland there was also an instruction, from the very start of the civil registration of death in 1855, that the doctor who had attended the deceased during their last illness should complete a medical COD certificate to be sent to the registrar.^12^ Although this was a legal requirement, if there had been no medical attendant a COD offered by the lay informant would often be entered into the death register instead.^13^ In the small minority of deaths that were suspicious or accidental, the case was referred to the procurator fiscal (equivalent to a coroner) who assigned a cause.^14^

In Scotland therefore, the recorded causes of death might be linked to more general patterns of medical attendance.^15^ When a person fell ill or met with an accident, the likelihood of being attended by a medical professional (who might later use their symptoms as a guide to a COD) was affected by the local availability of qualified individuals. This in turn depended on population density and terrain as well the ratio between doctors and people. It might have been necessary for a family to weigh up the cost of summoning a medical attendant and their household resources against their assessment of the likely benefits of calling the doctor. The latter may well have been influenced by the age, gender, and existing infirmity of the patient.^16^ The nature of the disease itself might have played a role, with acute conditions more likely to induce death before a doctor could be called.^17^ Previous analysis has shown that together these factors meant that between the late 1850s and 1870 fewer than 20 per cent of causes of death in some rural parts of Scotland were certified by a doctor, compared with around 90 per cent in some towns.^18^ Levels of certification improved with time, and this will have influenced the range and character of reported causes of death and the balance between different causes.

In some countries, those certifying a death had to choose a cause from a prescribed list. For example, in Norway, a list of 122 causes was introduced in 1861, which effectively standardised the terms appearing in the death registers.^19^ Although the Registrars General for Scotland (with the help of the Royal College of Physicians in Edinburgh) laid out nosologies that they hoped doctors would follow, and provided guidance on phrases they wanted doctors to avoid, this did not have the effect of standardising the terms appearing in the death register.^20^ Doctors were more likely than lay informants to offer a scientific cause, but the range of conditions and the way they were described by doctors varied considerably over time and between individual doctors. The causes offered will have been affected by changes in disease knowledge, fashion, and by the opinions of both the medical establishment and the statistical authorities about which causes were acceptable.

Medical theory and new knowledge about disease causation were developing rapidly over the nineteenth and twentieth centuries. Awareness of the causal agents and mechanisms involved in the spread of particular diseases was growing, and this was reflected in changes in definitions and terminology used by those reporting deaths such that symptomatic causes (such as ‘consumption’) were superseded by more precise definitions (such as ‘pulmonary tuberculosis’), together with a move to greater precision in diagnosis. The considerations occupying the statistical authorities differed slightly from those occupying the medical establishment. While doctors were concerned with medical and aetiological precision, the statistical authorities wanted to ensure that every COD was placed in an appropriate category and were often concerned about doctors’ ability to distinguish different causes that might present in similar ways. The issue was even greater for causes offered by lay informants: W.P. Dundas, Registrar-General for Scotland, noted in his second Detailed Annual Report, covering 1856, that he had had to include all infant deaths from ‘bowel hives’ in the ‘un-specified’ category because when informants from ‘the lower classes in this country’ reported this COD they could mean any one of three conditions: ‘first Fits or Convulsions; second Inflammation of the Bowels, or Enteritis; and third, Diarrhoea’.^21^ Reid et al. have described how the Scottish statistical authorities changed their views on the acceptability of the term ‘old age’ as a COD over time, and the way that doctors differed in the speed with which they replaced ‘old age’ with more precise medical disease terms.^22^ This example of a more widespread reduction in the use of vague and symptomatic terms for causes of death indicates that an apparent rise in a COD such as cardiovascular disease might actually represent a change in recording practice.^23^

This means that it is not always clear whether a difference over time and place really represents a change in mortality rates from a particular disease (affected by either disease prevalence or vulnerability to the condition), or whether it reflects increasing medical knowledge, changes in medical attendance, or diagnostic fashion: what Alter and Carmichael evocatively described as ‘local physicians insinuate[ing] their knowledge in humdrum settings’.^24^

The analysis of individual level causes of death is also affected by the increasing complexity of causes returned by medical professionals over time. Reid et al. show that on the Isle of Skye and the town of Kilmarnock, in the Scottish counties of Inverness-shire and Ayrshire respectively, the percentage of deaths where more than one cause was offered increased from around 10 per cent in the 1860s to nearly 30 per cent by 1900.^25^ This introduces the possibility of variation in whether the proximate or underlying COD was reported first, and when only one COD was reported, whether that was in fact the proximate or underlying cause. From the late nineteenth-century, until 1964, doctors in Scotland were supposed to record the primary (i.e. underlying), COD first on the certificate.^26^ There were labelled spaces for ‘primary’ and ‘secondary’ causes on the certificates, but these labels were not retained when the causes of death were copied into the death registers. There is evidence that even doctors were confused about whether the primary cause was the one which occurred earliest chronologically, or was the one which finally carried off the deceased (the proximate cause), with phrases such as ‘acute pneumonia following measles’ appearing in the register instead of ‘measles followed by acute pneumonia’.^27^

This is then linked to the issue of how causes of death were treated by the statisticians. Given that doctors were supposed to list the underlying cause first, can we assume that contemporary clerks allocated the death to a grouping represented by that first-listed cause? Or did they do as coders should do today, and take an assessment of everything on the death certificate to determine how to allocate the death to a higher category within a nosology? Unfortunately, it has not been possible to find records giving details of the classification process in Scotland that would shed light on these practices.

This leads us to the issue of how researchers have coped with the way that the published causes of death change over time. There has been much excellent work discussing the best way to deal with the thorny issues of (1) conditions which are moved between very different categories, and (2) the changing proportion of unknown or ill-classified deaths over time.^28^ These issues will not be rehearsed here, at least partly because the use of individual level causes of death avoids many of these problems. For example, with individual level data it is possible to identify which deaths were said to be due to typhus and which to typhoid, circumventing the need to aggregate them to match some classifications in which they were grouped together. Nevertheless, most users of individual level causes of death will want to compare the distribution of causes in their sample with those in the published tables, and to do this over time. This necessitates consideration of a much larger range of COD strings and their possible meanings, which requires considerable prior knowledge about what exactly historic terms mean, and—if they change meaning over time—when that shift took place. This knowledge (particularly regarding the timing of changes in the use and meaning of certain medical terms) can be significantly advanced by detailed analyses of the causes of death themselves. Observation of how terminology, usage and medical attendance evolved over time can extend our understanding of why certain changes in recorded causes of death took place.

This produces a chicken and egg situation: we need a classification to help analyse large collections of individual causes of death, but any classification depends on knowledge about the meaning of reported causes of death which may be changed or qualified by the results of analysis using that classification. The solution is to employ a flexible coding and classification system which includes an intermediary stage of tidying and coding specific words or phrases, rather than coding them to conditions we think (or even know) those words or phrases represented. The codes assigned in this way can then be built up into a range of different classifications suitable for different circumstances. Furthermore, they can then be easily re-classified once more knowledge of the way doctors were using them is gained.

The next section outlines previous approaches to the classification of historic causes of death and describes how our approach differs.

## Previous approaches to individual historic causes of death and justification of our approach

**Figure 1.**
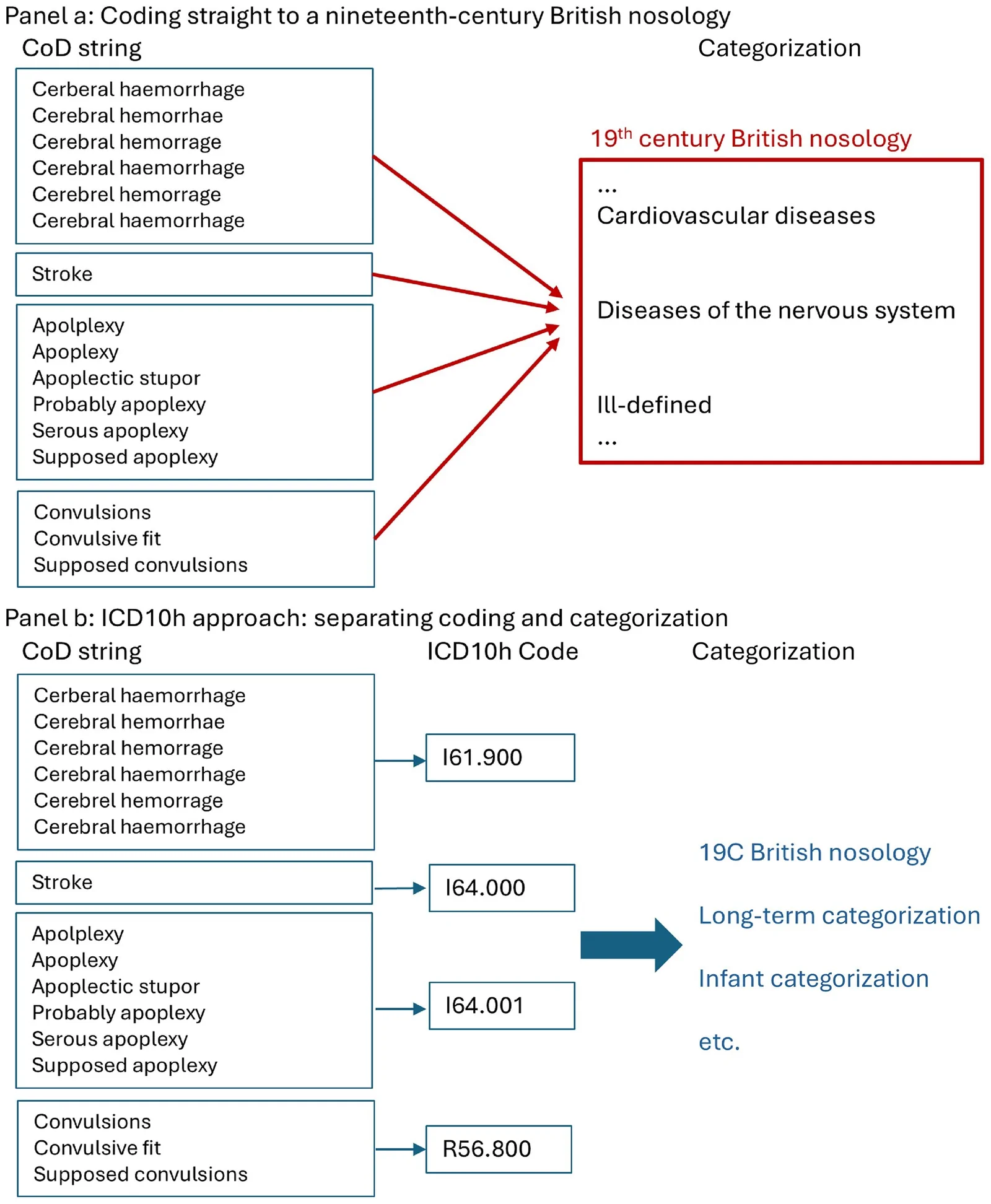
Different approaches to individual-level causes of death (COD).

Previously, those working with individual level causes of death have allocated each COD string (or cleaned string) straight into one of a small number of COD categories. These categories are often based on a classification or nosology contemporaneous with the period being studied, to enable comparisons with published data for the wider geographic area from which the data in the (usually smaller) dataset was drawn. Some authors adapt contemporary classifications or devise their own to enable particular causes of interest to be singled out; for example, the BeRaSaRo-system designed by Bernabeu-Mestre et al., which was based on the classifications used by Jacques Bertillon in 1899 and by Thomas McKeown in 1976, the latter itself based on nineteenth-century British nosologies.^29^ Panel a of figure 1 demonstrates this approach. Original COD strings, complete with mis-spellings, are grouped into four tidier terms, depicted by the boxes on the left of the figure: cerebral haemorrhage, stroke, apoplexy, and convulsions. Following a nineteenth-century British nosology, all these four terms can then be placed in the ‘diseases of the nervous system’ category. While this has been a successful strategy for use with small, time-limited (generally nineteenth-century) datasets, it has several limitations that make it difficult to compare results for different places or long time periods.

Firstly, such approaches prevent flexibility as they necessitate a blanket decision that a specific cause belongs in a particular group. This can be problematic because, as the discussion above has indicated, the use of particular causes changes over time, and may vary from place to place. Secondly, such categorisations are often good for comparisons over short time spans but make it difficult to compare over long time spans or between places with different death coding contexts. This is particularly the case when it is desirable to compare causes in a dataset with those in a published table or tables. The data for which we designed our new system stretches from 1855 to 1973—a period of massive change in the way that causes of death were conceived, recorded and classified. While a nineteenth-century British classification might work well with the nineteenth-century part of our data, and the ICD10 system would be suitable for the final decades, neither are appropriate for use over the entire time span. We need a system which will allow us to both consider long-term changes and compare short periods with contemporaneously published data. A single classification will not do this. Thirdly, researchers are given no precise instructions about how to allocate individual messy and complicated causes into the higher-level categorizations. For example, ‘phthisis’ referred to respiratory tuberculosis, but when ‘abdominal phthisis’ was written on a death certificate should this be treated as ‘respiratory tuberculosis’, ‘abdominal tuberculosis’, or something different? This is a particular issue when dealing with vague terms, which may represent a variety of different conditions.

Some have tried to deal with these issues by classifying to more than one scheme; for example, Revuelta et al. code both to BeRaSaRo and to ICD10.^30^ However, coding large volumes of individual strings is time-consuming. The solution that we chose, illustrated in Panel b of figure 1, is a flexible, detailed, and highly granular coding system where individual causes, and distinguishable variations of these, are each allocated a code. These codes can then be built up into a variety of different categorisations.

We based our system on ICD10 because the level of detail in the latter means that it is suitable for use at the later end of our period and could relatively easily be adapted to encompass historic causes (the opposite would not have been possible as historic nosologies were much simpler than ICD10). We adapted ICD10 both by adding over 3,500 additional codes for causes only seen in the historic records and by providing alternative ways of grouping the causes, which are more suited to historic eras.^31^ Before we provide details of our own system, therefore, it is helpful to outline the ICD10 system itself.

ICD10 is a highly granular system, providing over 14,000 distinct codes for diseases or CODs.^32^ These causes are grouped into twenty-two chapters, each referring to a high-level COD category. Most chapters are associated with a letter of the alphabet in a one-to-one correspondence, although some letters represent more than one chapter and some chapters cover more than one letter. The letters form the first part of the disease code, followed by two digits that refer to a more precise disease group within that chapter. Most codes then have a decimal point, followed by a further digit which indicates varieties of conditions within the disease group. For example, Chapter 1, Certain Infectious and Parasitic Diseases, includes codes from A00 to B99. Within this chapter, A00 refers to varieties of cholera, A01 to varieties of typhoid and so on. A00.0 and A00.1 refer to variants of cholera due to different biotypes of the cholera bacterium, and A00.9 refers to cholera that has not been specified as due to a particular biotype.^33^ This last code follows the principle laid out in 1864 by James Stark, Scotland's first Superintendent of Statistics, that in a statistical nosology there should always be a ‘not further specified’ option within each category.^34^ Such codes for diseases that are named but ‘not further specified’ tend to be the most widely used codes when dealing with historic contexts.

ICD10 therefore contains elements of both ‘coding’, in that it assigns a specific code to a particular cause at a granular level, and ‘categorisation’, in that it groups those codes and causes into a much smaller number of categories comprising similar causes. These categories can be assembled into larger classes for ease of analysis. Most coding systems are part of a classification system; however this does not mean the codes have to be transformed into the in-built classification—they can be grouped in other ways, and we use this principle in our new system, as outlined in the next section.

## An overview of ICD10h

ICD10h is a two-part coding and categorisation scheme based on ICD10. Where there is an exact match, a string is assigned to an ICD10 code. Additional codes (designated by additional digits) are introduced for historic terms within existing chapters (e.g., ‘consumption’ under ‘pulmonary tuberculosis’). These codes can then be built up into different categories and classes, for example to match existing nosologies, for specific age-groups, for particular time periods, or for international comparisons. Terms that change meaning over time (e.g., teething) are given the same code each time that specific term is encountered, but that code can then be allocated to the appropriate category for a particular era. Vague terms can be reunited with historically similar causes (e.g., ‘pleurisy’ and ‘congestion of the lungs’ with respiratory diseases). This section describes the principles of the coding process, and a later section will describe HistCat, one of the historic categorisations we have developed for use with ICD10h.

To better explain the system, we set out some important principles of ICD10h

ICD10h takes the four numerical digit ICD10 codes as a starting point (e.g., A00.9 for ‘cholera, un-specified’), and transforms them into ICD10h codes by adding two further zeros on the end. Therefore, A00.9 in ICD10 becomes A00.900 in ICD10h, and still refers to ‘cholera, un-specified’. In the few cases where an ICD10 code does not have a digit after the decimal point (e.g., A35 Tetanus), we add three zeros after the decimal point.

We give each COD string an ICD10h code ending 00 if there is an ICD10 code for that particular string (or a standardised version of that string) in ICD10. For example, we code ‘cholera’ to A00.900 and ‘stroke’ to I64.000. Importantly, we code the **word**, *not* our interpretation of what it meant in a historic context. In most cases, coding the word and coding our interpretation of it result in the same code; we assume, for instance, that the term ‘measles’ in the mid-nineteenth-century refers to the same disease as ‘measles’ in the early twenty-first century. The rationale behind this is that the same word or group of words always has the same code and the *classification* of that code can be adjusted to reflect any changes in the meanings of the word.

An example of where coding the word and our understanding of it do not lead to the same code is provided by ‘teething’. In the first half of the nineteenth-century, ‘teething’ allegedly killed a considerable portion of babies in Britain. Out of the 53,157 infant deaths occurring in England and Wales in 1851, for example, 4,400, or 8.3 per cent, were recorded as being due to teething.^35^ Although it is generally assumed that the historic COD ‘teething’ is an indicator of diarrhoea, in the spirit of ‘coding the word rather than the interpretation’ we code it to the ICD10 placement of ‘teething syndrome’ (K00.7) even though that is under ‘diseases of the oral cavity’, distinguishing it by adding a historic suffix so it becomes K00.704.^36^ We then deal with this in our coding system by grouping it with diarrhoeal diseases or another group as appropriate for the historical context.^37^ Keeping terms separate, rather than aggregating them into a code with other terms, allows trends in the separate conditions to be examined to determine whether deaths from one cause always closely mirror deaths from another cause in different places and at different times.

Where we come across an historic term that does not match exactly to anything in ICD10 (we refer to such terms as ‘archaic’), we create new codes in an appropriate part of the nosology (using historic evidence to guide our interpretation of the appropriate location) using the additional two digits added to the original ICD10 cause.^38^ For example, we interpret ‘apoplexy’ as ‘stroke’ and therefore we adapt the code for ‘stroke’ (I64.000) to create a code for ‘apoplexy’ (I64.001).^39^ This allows us to examine the changes from the use of historic/archaic terms to more scientific terms over time, as well as the potential for some categorisations to allocate apoplexy to a different categorisation if a setting is discovered where it does not mean ‘stroke’.

We also create new codes where we suspect a cause changed meaning over time, to allow the ‘new meaning’ to be separated from the ‘old meaning’. For example, the term ‘choleraic diarrhoea’ was frequently used in nineteenth-century Scotland, but we did not know whether it really represented cholera or diarrhoea. We therefore created a new code, A00.901, for it as a subcategory of cholera (A00.9). In most cases, these will be grouped together, but the separate code allows us to examine and further understand how it was used.

Cancer is the one causal group that is treated slightly differently in ICD10h compared with ICD10. ICD10 chapter II (Neoplasms) covers letters C and D, with C reserved for neoplasms confirmed as malignant using laboratory tests, and D covering ‘benign neoplasms’ as well as a small number of codes for neoplasms of ‘uncertain or unknown behaviour’. It would have been rare for a nineteenth-century cancer diagnosis to have involved a laboratory test, and even if it was, this is unlikely to have been mentioned on the death certificate.^40^ Therefore, if coded using ICD10, all nineteenth-century cancers should be placed in chapter D. We decided to take a different course of action in ICD10h and code our nineteenth-century cancers to chapter C. If we had not done this, we would have needed to make a large number of new codes in chapter D in order to contain our historic cancers in similar detail to modern ones as ICD10 chapter D contains rather few codes for cancers of uncertain origin and offers a smaller number of parts of the body where cancers can occur. We took the view that any cancer recorded as contributing to a death was malignant, unless the certificate expressly stated otherwise. However, we did create separate subcategories under each code for the main terms for cancer which occur in the early civil registers: cancer, scirrhus, sarcoma, epithelioma, tumour, malignant disease, and carcinoma, which receive codes .x01 to .x07 respectively. In addition, .x00 is used if the modern term ‘(malignant) neoplasm’ is given; .x08 is reserved for any other specified form of cancer/neoplasm. For completeness, we have provided such terms for all parts of the body, even although the type of cancer may now be known not to appear in particular sites.

## Creating ICD10h

To build the ICD10h codes, we concentrated on creating a dictionary of new codes for archaic causes, using two sources. Our first dataset consisted of COD strings for the 20,115 deaths on the Isle of Skye and the 23,715 deaths in the Scottish town of Kilmarnock, 1861–1901.^41^ Our second was 22,024 COD strings relating to 93,000 deaths on the island of Tasmania 1838–1899.^42^ These sources were chosen because they were most relevant to the Scottish data for which the ICD10h was designed. Although the Tasmanian data expand the range of possible causes outside the context of northern Britain, the range is still anglophone and in the nineteenth-century many doctors in the antipodes, including those born there and those emigrating, were trained in Britain—often in Scotland, which had the largest medical schools.^43^ We did not have access to any COD strings for the twentieth-century period, but we assumed this period would be covered by the combination of nineteenth-century terms and the more modern detailed causes captured by ICD10.

The following steps had already been taken by the researchers who produced the original data: all strings were parsed so that separate causes of death were placed in different fields (for ‘first cause listed’, ‘second cause listed’, ‘third cause listed’, etc), and details of the length of the last illness (included in the Scottish data) separated out. For example, the certificates contained a column headed ‘Cause of Death, Duration of Disease, and Medical Attendant by whom certified’, and an example of text in this column is: ‘Enteric Phthisis 2 years Pulmonary Phthisis 1 year As cert by [name of doctor]’. This had first been entered into three fields, one for the causes of deaths (here ‘enteric phthisis and pulmonary phthisis’), one for duration of disease (here 2 years, 1 year), and one for the name and qualifications of the doctor. The parsing process separated out the two elements of the COD, placing ‘enteric phthisis’ into a field for the first cause and ‘pulmonary phthisis’ into a field for the second cause. Lists of the first, second, third, etc causes were combined and a further list of unique COD strings for single causes was produced. These strings were then tidied by eliminating spelling variations, removing extraneous spaces, and other standardisation. This standardisation took a slightly different form in the two datasets, so there was still some variation. Unique tidy causes for both datasets were combined to give a total of 19,741 historic strings of single CODs. These CODs were hand coded to ICD10h, with the help of the online ICD10 search tool.^44^ To understand more esoteric terms, we turned to historic nosologies, such as those provided in the Registrar-General's reports for England (and Wales) and Scotland, nineteenth-century medical texts,^45^ and more recent histories of disease.^46^ New terms were created during the coding process and reviewed at the end.

We then automatically allocated codes to each of the CODs reported for an individual, so that individuals had as many codes as they had causes of death. Because some multiple CODs have their own ICD10 codes (e.g., B05.2 Measles complicated by pneumonia), we inspected all multiple causes and assigned a single code to reflect this, replacing ICD10h codes where necessary (e.g., for complications of some other common infectious diseases). For multiple causes where more than one code was retained, we allocated an underlying cause (the disease or injury that initiated the train of morbid events leading directly to death) following ICD10 rules, which involve ignoring symptoms and trivial conditions and ordering the other causes by a plausible temporal sequence.^47^ We used a version of NCHS's ACME (automated classification of medical entities) software to assign underlying causes based on the ICD10 codes associated with the ICD10h code; however, the software was unable to deal with a significant proportion, and these cases were resolved manually.^48^

A manual, which both outlines the system and gives instructions for coding new individual level historic datasets, is available, as is the full list of codes and the list of historic strings used to create the system.^49^

## The HistCat classification for long-term comparisons

The availability of individual causes of death has been hailed as a way of circumventing the problems of nosological changes. To some extent this is true, but there is no getting away from the desirability of comparison with published schema, and it is therefore important to create look-up tables for at least some published nosologies. Because the project data stretched over 150 years, we wanted to enable a comparison of the numbers of deaths observed in our data with the numbers published using contemporary nosologies. We therefore generated a classification system based on the most detailed Scottish nosologies published over the whole time period. We aggregated these up to maintain useful but distinct categories, combining categories where the more detailed categories in the Scottish nosologies were affected by transfer between categories over time. More detailed descriptions of the process can be found elsewhere.^50^

The categorisation was created using published data, and it is generally straightforward to allocate individual deaths into the categories used. However, a major issue emerged with deaths described as due to ‘debility’ or ‘weakness’. The published nosologies had categories for ‘atrophy and debility’ under the general heading of ‘perinatal causes’, and ‘senile debility’ under ‘old age’. Sixty-one per cent of the deaths attributed simply to ‘debility’ in the Skye and Kilmarnock data occurred among those aged 60 or over (43 per cent occurred in those aged 70 and over), and 25 per cent in those under age one. We presume that the Registrars General also used age to allocate COD. We also recommend doing this, but we needed a solution for allocating strings to categories in the absence of age. We therefore created a ‘debility’ category, and then reallocated these deaths based on age: those under age one to perinatal causes, those age 70 and over to old age, and the remainder to ill-defined.

HistCat codes are provided on the ICD10h Masterlist.^51^ The European team working on ICD10h has also created a separate categorisation for infant mortality, and is working on other classifications for different ages and to match other historic classifications.^52^

## Applying ICD10h to Scottish COD strings, 1855–1973

The ICD10h scheme was borne out of the need to code and categorise the individual-level causes of death produced by the Digitising Scotland (DS) project. This project transcribed all the individual-level information contained in the civil registers of birth, marriage, and death for Scotland from 1855 to 1973, and will merge this data with the already digitised information on vital events from 1974 to the present day.^53^

The DS database contains information on COD from around eight million deaths records (8,290,642), which were transcribed as seen. Separating out multiple causes using characters which indicated CODs had been written on different lines resulted in a full set of 12,338,989 COD strings, although many of these still contained multiple causes. A training dataset was created by discarding any strings with unreadable characters, selecting a random subset from the 11,995,358 remaining strings, and eliminating duplicates. This subset of 92,222 unique strings were then hand-coded to ICD10h by a team who were instructed to correct spelling errors and split any remaining strings containing multiple causes (assigning a code to each).

These manually assigned codes were used to automatically code the remaining strings as follows.^54^ Strings that were identical to a manually coded string—possibly with changes to whitespace—were assigned the corresponding code(s). Next, spelling correction (allowing an edit distance of one) was applied using a dictionary derived from the manually coded strings as corrected by the coders, and a match was again attempted. The remaining strings were passed through a rule-based splitter to separate multiple causes within a single string, and to remove labels such as ‘(a)’ and durations of last illness; matching was then attempted on the parts. This left 6.7 per cent of the strings unmatched, and a further 2.1 per cent with some of their parts unmatched. These were passed to a Naive Bayes statistical matcher, trained on the manually coded strings (weighted according to their frequency in the full corpus). Of these, 0.6 per cent could not be assigned a code because they contained no words that appeared in the manually coded data, or were rejected by the splitter as having no useful words.

Although ICD10h codes have been applied to all parsed causes, underlying causes have not yet been allocated for the DS data. However, if the first recorded cause was in the ill-defined HistCat category, we looked to see if there was a cause for that individual that was not ill-defined and took the first of those to be listed. An assessment of this procedure is provided below.

In the rest of our paper, we illustrate the uses of ICD10h using examples drawn from Skye, Kilmarnock, and the DS database.

## Using ICD10h: comparisons with published data

**Figure 2.**
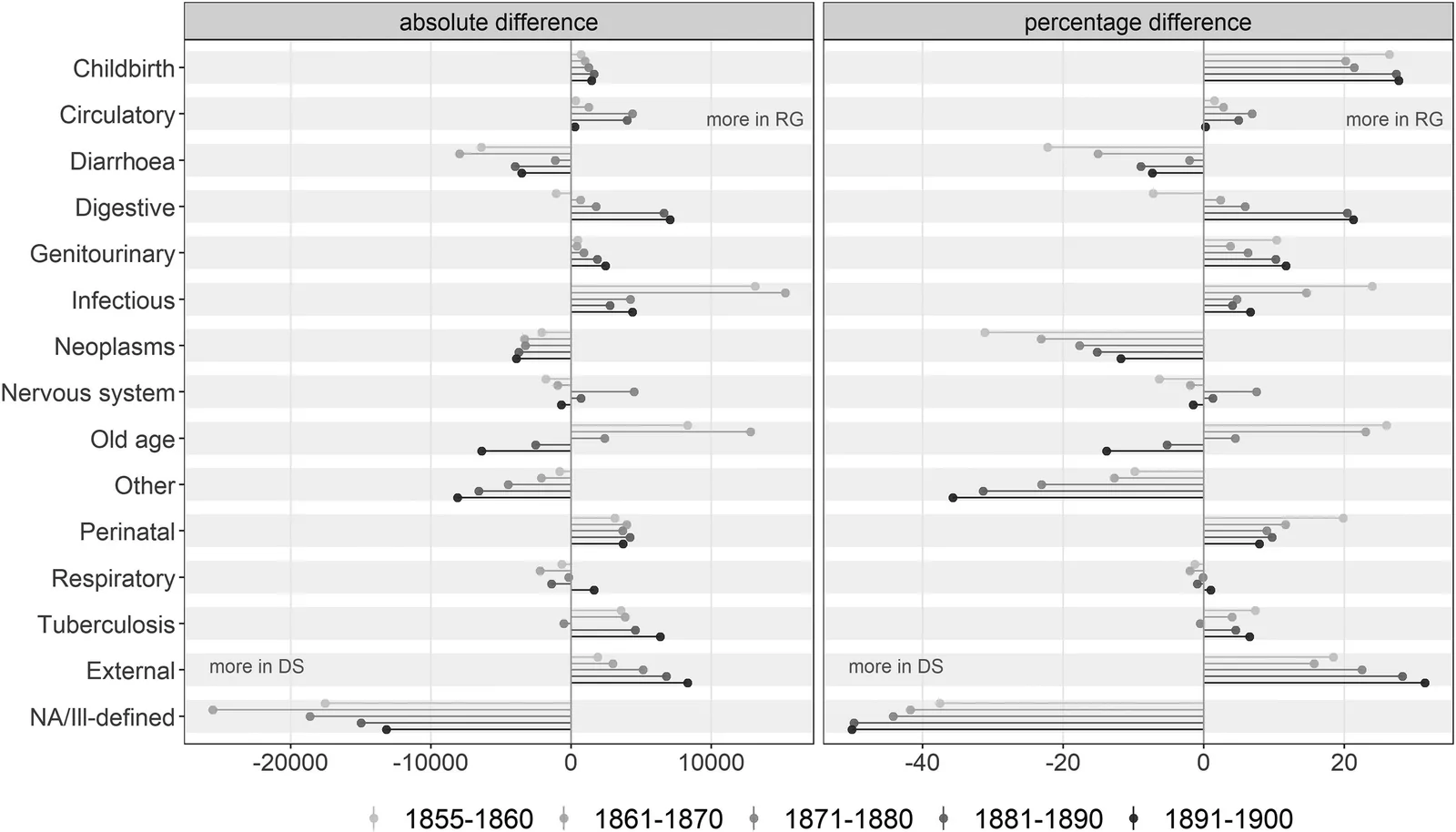
Comparison between deaths in Scotland coded to HistCat categories in Digitising Scotland (DS) data and published sources (RG), by decade. Sources: Digitising Scotland data; R.J. Davenport. ‘Annual deaths by cause, age and sex in Scotland, 1855–1949’, https://www.campop.geog.cam.ac.uk/research/projects/longrundeathcauses/ (2012).

First, we assess the performance of the scheme by comparing the COD distributions of the DS data with those from the published sources, both of which have been coded to HistCat. Figure 2 shows discrepancies between the numbers of deaths coded to each category in DS and in the published sources, by decade. The left-hand panel shows the absolute differences, and the right-hand panel shows these differences as a percentage of the figures published by the Registrar General. In both cases, bars on the left indicate that more deaths were coded to that category in the DS dataset, and bars on the right indicate that more were allocated to that group in the published sources.

It is clear that DS allocated more deaths to the NA/ill-defined and other categories, probably due to readability, transcription, and parsing issues. Nearly all the excess DS deaths in the NA/ill-defined category are accounted for by NA, that is, they were not allocated a code at all, and this is particularly true for the earlier decades. It is likely that more uncommon strings and complex descriptions remained uncoded, and this may contribute to increasing under-coding of external deaths over time, as such deaths tend to be described at length and with great variety. Other differences might be due to the fact that DS used only the first recorded non-vague cause, and this is particularly likely to have led to an undercount of deaths due to childbirth, and to the change from under-recording to over-recording of old age over time. Categories across the causal spectrum see deficits in DS deaths, with notable exceptions of neoplasms and diarrhoea, where DS appears to over-allocate deaths. The latter could be related to the fact that diarrhoea was frequently recorded with other diseases and the Registrar General may have assigned such deaths elsewhere. There could also be some CODs, particularly common in later decades, coded by the Registrar General to digestive diseases but by DS to diarrhoea. The excess in cancer deaths appears, on investigation, to be an artefact of the machine learning (ML) algorithm used for a small proportion of difficult-to-code textual descriptions. The Naïve Bayes ML approach associated common phrases within a COD, such as ‘of the’, to cancer because that was the most frequent code associated with that phrase. This meant that this phrase was coded into a cancer code irrespective of the words around it, for example ‘scald of the’.

Comparison of the absolute and percentage differences indicates that although some causal groups, such as maternal mortality, appear to be only marginally affected, they can show large percentage discrepancies, and those interested in studying the evolution of particular causal groups and terms over time are advised to use the original strings to search for and identify relevant causes more securely.^55^ Nevertheless, these categories form relatively small proportions of the total, so the distribution of deaths is far less affected (see Appendix Table). The next section explores some of these differences in further detail using data from the town of Kilmarnock in Scotland.

## Using ICD10h: coding and categorization effects

**Figure 3.**
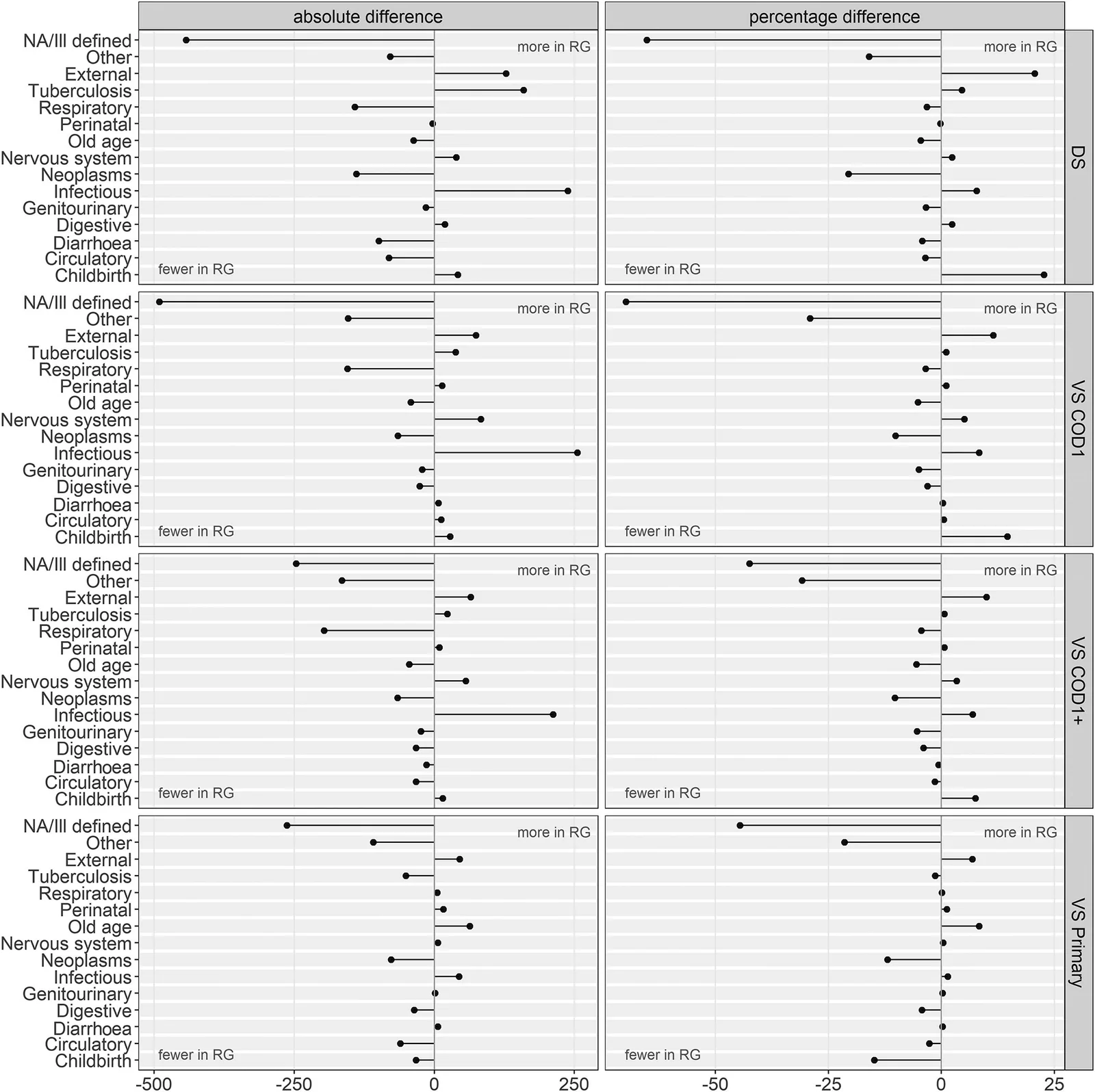
Coding and categorization comparisons: Kilmarnock 1861–1901. Sources: Digitising Scotland dataset; Victorian Scotland dataset; Registrar General for Scotland's Detailed Annual Reports, 1861–1901. Notes: This graph compares, for Kilmarnock, the numbers of deaths in different categories published in the Registrar General's Annual Reports (RG) with numbers in DS (Digitising Scotland dataset), and in VS (Victorian Scotland dataset) taking three different approaches to deaths where more than one cause was listed: VSCOD1 categorizes deaths according to the first cause listed; VSCOD1 + takes the first cause that is not ‘ill-defined’; and VSPrimary allocates a primary or underlying cause following ICD10 guidelines.

To try to disentangle the different effects of transcription, automatic coding, dealing with multiple causes, and different categorisation practices, we make use of three different data sources relating to deaths in the same Scottish town between 1861 and 1901. Ultimately, they all derive from the same death registers, but they have been transcribed, coded and categorised differently. In figure 3, DS refers to DS as described above, with deaths transcribed, automatically coded, and categorised using the first listed cause that was not ill-defined. We compare this to an earlier transcription of the same registers made by the ‘Demography of Victorian Scotland’ project (indicated by VS). These causes of death were hand-coded to ICD10h and categorised in three different ways: taking the underlying or primary cause (primary), taking the first listed cause (COD1), and taking the first listed cause that was not ill-defined (COD1+). We compare each of these to the figures published in the Registrar General's annual reports, for which we have, as explained above, rather little information on the way they were actually coded. The left-hand column of figure 3 provides the absolute difference in the numbers of deaths allocated to each causal group over the 40-year period and the right-hand column gives the percentage difference. In each comparison, negative numbers indicate more deaths in the source indicated by the panel on the far right and positive numbers indicate more in the Registrar General's annual reports.

The graphs indicate that for DS, the same patterns occur in the much smaller sample of the town of Kilmarnock as for Scotland as a whole. Comparison between the various ways of coding the VS Kilmarnock data can shed some light on the drivers of some of these patterns.

Firstly, the fact that DS has more ill-defined than VS COD1+, despite identical coding procedures, suggests that the volume of DS data and automatic coding might have introduced additional issues. It is possible that the relatively large number of causes with unreadable characters made such deaths impossible to code accurately, increasing the numbers and percentages of deaths in the NA/ill-defined category. Unreadable characters may have been particularly prevalent in external causes, which often contained place names.

Identifying and using the primary cause to allocate deaths to a causal group produces the greatest similarity to the figures published by the Registrar General. It seems probable that although doctors were supposed to list the primary or underlying cause first on the death certificate, whoever assigned each death to a category did not make a blanket assumption that the first listed cause was the primary one, and took a more careful look at all the recorded causes when assigning an overall cause. Using a more precisely specified cause (for the same death) if the first listed cause was ill-defined (VS COD1+) results in a considerable reduction in the numbers in the ill-defined category, but leaves certain systematic discrepancies, including an underestimation of infectious diseases and tuberculosis and corresponding overestimation of respiratory diseases, as well as an underestimation of maternal mortality (childbirth). Respiratory diseases such as pneumonia are common complications of infectious disease and doctors frequently wrote these on the wrong order on the death certificate. They might have made it clear using wording which was the primary cause (e.g., pneumonia following measles) but the parsing process used here did not use connecting words to re-order causes.

Maternal mortality is a numerically small but important cause, and the discrepancies (larger for DS than VS) are easier to see in the percentage graphs. Previous analysis has shown maternal mortality was often ‘hidden’ by doctors and the parsing process might also have contributed to this, for example by separating ‘peritonitis, difficult labour’ into first cause ‘peritonitis’ and second cause ‘difficult labour’—in this case peritonitis becomes separated from the information that a woman had recently delivered and has to be allocated a non-maternal code. Use of the primary cause both corrects this and picks up cases of maternal mortality not identified by the nineteenth-century clerks.^56^

## Using ICD10h: following terms and descriptions over time

ICD10h allows us to unpack broad COD categories and the diseases included within them. For example, the nineteenth-century death registers used to create ICD10h include a large number of terms that are considered to refer to tuberculosis, such as phthisis, pulmonary phthisis, abdominal phthisis, consumption, consumption of the brain, scrofula, scrofula of the bones and joints, scrofula of the skin, and so on. Although these are all generally accepted to refer to forms of tuberculosis, some authors suggest that the term ‘consumption’ may have been a more general term, used to describe a variety of different wasting diseases or even an illness such as pneumonia.^57^ In order that users are not constrained to classify consumption as tuberculosis, and can distinguish between uses of different terms for various manifestations of the disease, we allocated each of the terms ‘phthisis’, ‘consumption’, ‘scrofula’, ‘pulmonary consumption’, ‘pulmonary phthisis’, ‘pulmonary tuberculosis’, and ‘tuberculosis’ a specific historic code in ICD10h. Because we think they are most likely to refer to tuberculosis, these new codes are all placed within the existing tuberculosis categories, although it would be possible to extract them and place them elsewhere in a custom-made classification scheme.

**Figure 4.**
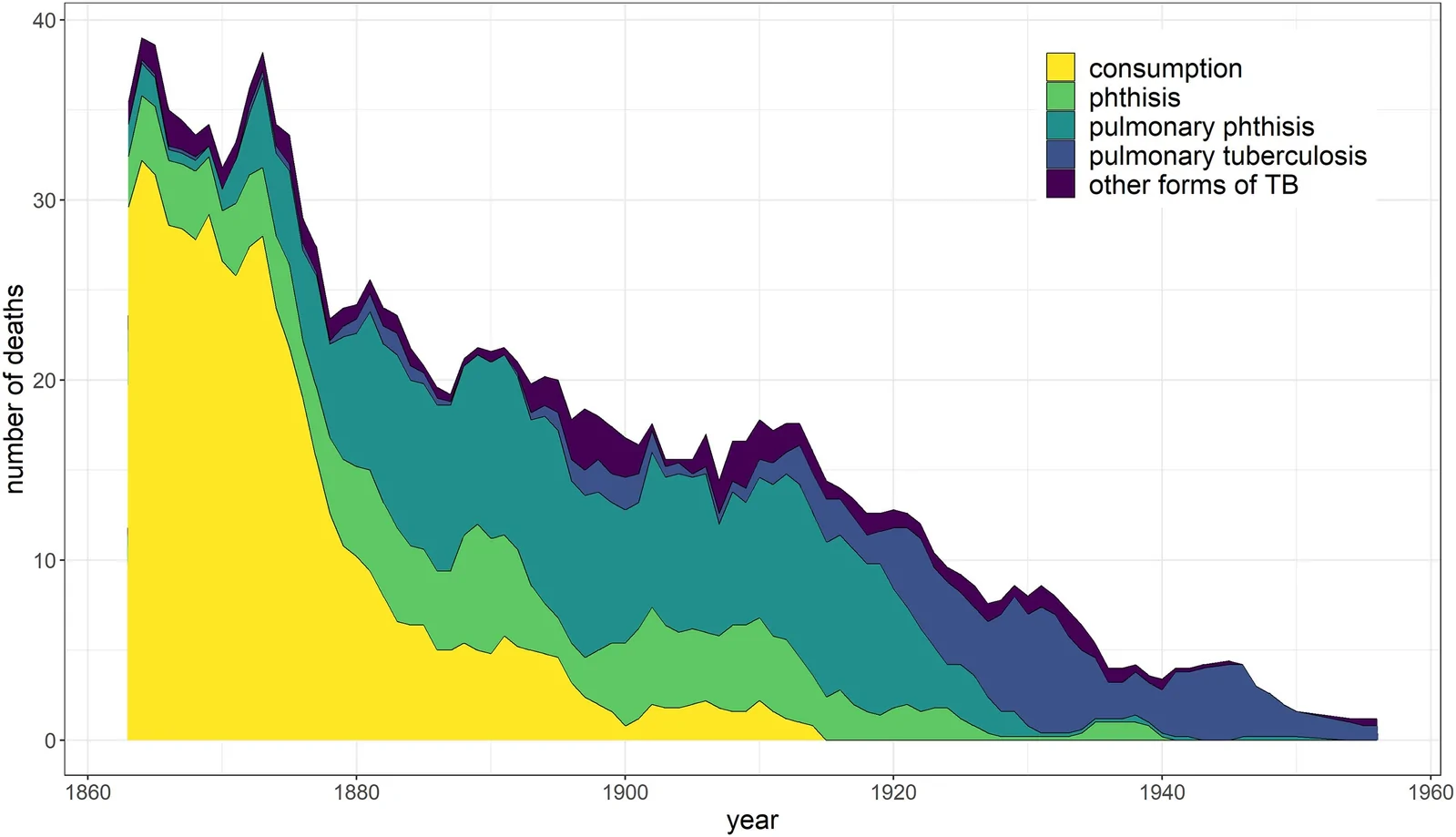
Deaths from various forms of tuberculosis on the Isle of Skye, Scotland, 1861–1959 (5-year moving average). Sources: VS dataset 1861–1899; Digitising Scotland Dataset 1900–1959. Note: ‘Other forms of TB’ include scrofula, tabes mesenterica, and cases of hydrocephalus stated to be tuberculous. ‘Consumption’ includes a few cases of ‘pulmonary consumption’, and ‘pulmonary tuberculosis’ includes a few cases of ‘tuberculosis’.

As figure 4 demonstrates, the specific historic codes in ICD10h allow us to see that there was a major step change in the reporting of deaths from tuberculosis on the Isle of Skye in the late 1870s when use of the general term ‘consumption’ was replaced by ‘phthisis’ and the more precise term ‘pulmonary phthisis’. The term ‘pulmonary tuberculosis’ was not employed in any great numbers until the 1920s. Anderton and Leonard demonstrate that a similar transition away from consumption occurred in Massachusetts, although tuberculosis replaced phthisis a decade earlier there.^58^ The figure suggests that deaths from tubercular disease declined steadily from the late 1880s, but it is possible that the marked downward trend between the 1870s and 1880s is related more to changes in reporting than to an actual decline in the disease. As the general term, ‘consumption’, was replaced by more specific terms, some cases may have been attributed to non-tuberculous causes; but further research is needed to confirm this.

## Using ICD10h: enabling comparative work

**Figure 5.**
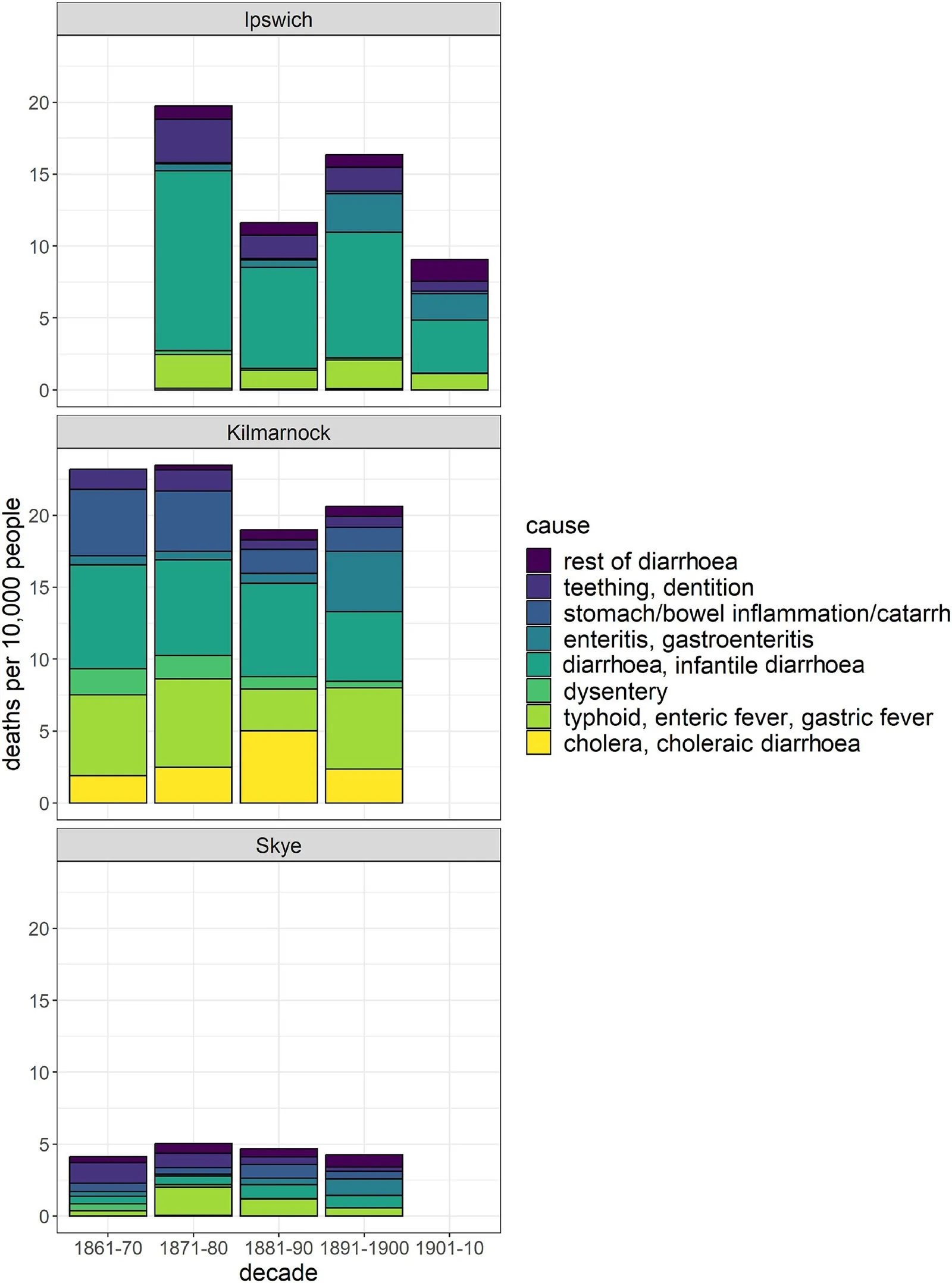
Mortality rates from diarrhoeal diseases; Skye, Kilmarnock and Ipswich, 1871–1910. Source: Skye and Kilmarnock data from the VS dataset; Ipswich data from E.M. Garrett et al., ‘Sociological Study of Fertility and Mortality in Ipswich, 1872–1910’, 2007. Rates calculated using the intercensal population average for each decade (Census Reports of Scotland and England and Wales, 1871–1911, www.histpop.org).

The ICD10h scheme has already allowed comparative work to be carried out within the SHiP project, when the causes of infant mortality were examined and contrasted across a selection of Europe's port cities.^59^ An example of the value of ICD10h for comparative work within Britain is presented in figure 5, which compares decadal rates of death on Skye and in the towns of Kilmarnock (Scotland) and Ipswich (England), from various diseases characterised by diarrhoea as a symptom. These conditions are often referred to as ‘filth diseases’ (cholera, dysentery, diarrhoea, and bowel diseases) as they arise from contaminated water or food and are frequently used as ‘flags’ of poor water, drainage, sanitation, or hygiene.^60^

The figure shows that Skye had a very low rate of deaths from diarrhoeal diseases, likely due to the rural setting which minimised the transmission of pathogens, while the two towns had higher rates. The situation in Ipswich improved over the period, with a decade-on-decade decline in rates interrupted by a resurgence in the 1890s—part of a national upswing in diarrhoea deaths across English towns associated with a series of long, hot summers.^61^ Kilmarnock exhibits a similar (although slightly muted) pattern of decline and resurgence. Despite these similarities, there were important differences in the range of terms used for deaths that could be broadly described as gastro-intestinal or fecal-oral.^62^ In Ipswich, diarrhoea was the predominant term used, with a much smaller number of deaths attributed to typhoid. Teething formed a relatively substantial proportion of deaths in the 1870s but gradually fell out of favour, whereas towards the end of the nineteenth century, doctors were encouraged to use enteritis and particularly gastro-enteritis for broadly diarrhoeal deaths.^63^ The same patterns of decrease in teething and increase in gastro-enteritis can be seen in Kilmarnock, but doctors there used a far broader range of causes than in Ipswich, including choleraic diarrhoea, typhoid fever, and inflammation of the bowels. The last of these causes is likely to be a non-specific synonym for diarrhoea, but the other two are identifiable diseases in their own right.

We are now in a better position to consider whether these differences in terminology reflect real differences and changes in the character and prevalence of disease, or simply changes in fashion and medical knowledge. The declines in archaic descriptions such as teething and inflammation of the bowels, and the rise in gastro-enteritis, are likely to represent the latter. It is more difficult to resolve the question of whether cholera and typhoid were really present in Kilmarnock but not (or far less) in Ipswich. The fact that Kilmarnock suffered peaks of deaths attributed to typhoid and diarrhoea in 1893 when there was a national outbreak and a peak in cholera deaths in 1882, again at the start of a national epidemic, suggests these reflected real patterns.^64^ Doctors could, of course, have been reacting to reports of these diseases elsewhere, but if so it is notable that doctors in Ipswich were not doing the same. A further clue is provided by comparison with the official counts produced by the Registrar General of Scotland. These attribute far fewer deaths to cholera than have ‘cholera’ or ‘choleraic diarrhoea’ written on the death certificate, and the annual numbers indicate that ‘choleraic diarrhoea’ must have been placed in the ‘cholera’ category up to and including 1880, but in the ‘diarrhoea’ category from 1881 onwards. This insight cautions against reading too much into the differences between the different diseases that present with diarrhoea as a symptom. Real trends and geographic differences in disease, exposure and risk during the nineteenth-century are likely to be best captured by large groupings, while fine comparisons can provide insight into medical knowledge and fashion, and certification and categorisation practices.

## Conclusions

Alter and Carmichael wrote that there is ‘no key’ to ‘translating archaic causes of death into modern terminology’—we do not disagree.^65^ The interpretations of archaic terms are likely to be contextual, place- and time-specific. They also wrote that ‘Continuity occurs at the level of individual deaths, not at the level of categories in a superimposed classification scheme’.^66^ We also argue that although individual deaths received very different treatment across time and between places, and are fiendishly difficult to deal with, they offer the best insight into changing COD patterns, and how those were recorded, categorised and understood, and we suggest that ICD10h is an important tool to help in this endeavour.

ICD10h will facilitate both micro- and macro-studies. At the micro-level, it will help both medical historians and historical demographers interested in particular diseases to probe their history beyond the restricted view presented by published tables of mortality statistics to offer insights into the aetiology of the diseases, the means by which they spread, the reasons for their decline, and how they were viewed and categorised by the medical authorities. It will also allow researchers to study all diseases, conditions or events recorded as contributing to a death, including conditions such as ‘shock’, ‘coma’, or ‘haemorrhage’ which, while relatively common, very seldom appear as the underlying COD as they are always brought on by a prior ailment or trauma. With ICD10h codes for such ‘multiple causes’, researchers can also observe how different causes inter-relate or tend to be associated with one another. The granularity of the system will make it easier for historical demographers to investigate and appreciate the ambiguity and situated nature of COD descriptions, and the ability to apply these granular codes to large datasets will encourage medical historians to see the continuities over time and place as well as the differences. Above all, it provides a mechanism for the insights of medical historians to be fed into the development of context-sensitive COD categorisations.

At the macro-level, the individual causes seen on historic death certificates can be allocated to a variety of historical and modern COD classifications, shedding light on broad trends and patterns and illustrating the way that changes in the death registration process in a particular setting may have created breaks and fluctuations in mortality trends from particular conditions. Importantly, we have illustrated how the system allows us to ‘unpack’ historic nosologies and registration systems, demonstrating them to be culturally and institutionally constructed entities, rather than ‘pure’ statistics.

ICD-10 has been translated into 42 different languages making international comparisons of causes of death much easier, and we are working with international colleagues as part of the SHiP/SHiP+/Great Leap projects to compile similarly informative international dictionaries of historical COD terms to be included in the ICD10h schema, enabling comparison of historic individual level causes of death across Europe, and hopefully beyond. With increasing numbers of databases that include large numbers of individual records detailing how people met their deaths, ICD10h offers a coding schema, and the means to build COD classifications, which will help researchers examine how the epidemiological and mortality transitions unfolded in unprecedented detail, without having to rely on the potentially distorting lens of changing official classifications and aggregated COD categories.

## Footnotes

1 A. R. Omran, ‘The Epidemiologic Transition: A Theory of the Epidemiology of Population Change’, *The Milbank Memorial Fund Quarterly*, 49 (1971), 509–38; T. McKeown, *The Modern Rise of Population* (New York: Academic Press, 1976); T. McKeown and R. G. Record, ‘Reasons for the Decline of Mortality in England and Wales during the Nineteenth Century’, *Population Studies*, 16 (1962), 94–122.

2 G. Alter and A. Carmichael, ‘Reflections on the Classification of Causes of Death’, *Continuity and Change*, 12 (1997), 169–73.

3 S. J. Kunitz, ‘Premises, Premises: Comments on the Comparability of Classifications’, *Journal of the History of Medicine and Allied Sciences*, 54 (1999), 226–40, 227, 239.

4 D. L. Anderton and S. H. Leonard, ‘Grammars of Death: An Analysis of Nineteenth-Century Literal Causes of Death from the Age of Miasmas to Germ Theory’, *Social Science History*, 28 (2004), 111–43, 138.

5 Kunitz, ‘Premises, Premises’, 240.

6 G. B. Risse, ‘Cause of Death as a Historical Problem’, *Continuity and Change*, 12 (1997), 175–88, 186–7.

7 ICD10h is a research tool created to facilitate the study of historical cause of death records and should not be used for any official purpose. It is based on the International Classification of Diseases, 10th Revision (ICD-10) version 2016 (Geneva: World Health Organization, 2016) but is not a recognised version or extension of ICD-10 and is not authorised by WHO. However, we have consulted with WHO: they recognise that ICD10h is a useful academic methodology and have not raised any objections to its creation. Data coded using ICD10h are not directly comparable with data coded in ICD-10, and the underlying or primary cause of death derived using the ICD10h methodology may be different from the underlying cause derived in ICD-10 according to the WHO rules. Note that ICD-10 version 2016 is not the most recent version of ICD-10; and that WHO now recommend the use of ICD-11; a more advanced and detailed classification.

8 ICD10h has been under development by the authors of this paper since 2012 for application to Scottish data. However, we have more recently teamed up with European colleagues in the Study of Health in Port Cities (SHiP and SHiP+) and the Great Leap network, to refine and extend the system to allow causes of mortality in a variety of languages to be compared and contrasted across European nations and beyond (see A. Janssens, ‘Constructing SHiP and an International Historical Coding System for Causes of Death’, *Historical Life Course Studies*, 10 (2021), 64–70).

9 A. Reid, E. Garrett, C. Dibben and L. Williamson, ‘A Confession of Ignorance’: Deaths from Old Age and Deciphering Cause-of-death Statistics in Scotland, 1855-1949', *The History of the Family*, 20 (2015): 320-44; A. Reid and E. Garrett, ‘Doctors and the Causes of Neonatal Death in Scotland in the Second Half of the Nineteenth Century’, *Annales de Démographie Historique (Paris)*, 1 (2012), 149–79; A. Reid and E. Garrett, ‘Medical Provision and Urban-Rural Differences in Maternal Mortality in Late Nineteenth Century Scotland’, *Social Science & Medicine*, 201 (2018), 35–43; A. Reid et al., ‘A Century of Deaths, Scotland 1855–1955: A View from the Civil Registers’, *Death in Modern Scotland, 1855–1955: Beliefs, Attitudes and Practices* (Bern: Peter Lang, 2016), 131–60.

10 B. Revuelta-Eugercios et al., ‘Older Rationales and Other Challenges in Handling Causes of Death in Historical Individual-Level Databases: The Case of Copenhagen, 1880–1881’, *Social History of Medicine*, 35 (2022), 1116–39.

11 Ibid (note 14).

12 These certificates no longer survive.

13 Select Committee on Death Certification, *First and Second Report from the Select Committee. Proceedings, Minutes of Evidence, Appendix and Index.* (1893).

14 R. S. Shiels, ‘The Investigation of Sudden and Accidental Deaths in Mid-Victorian Scotland’, *Death in Modern Scotland, 1855–1955* (Bern: Peter Lang, 2016), 177–94.

15 Reid and Garrett, ‘Doctors and the Causes of Neonatal Death’; A. Reid and E. Garrett, *Influences on the Medical Certification of Child Deaths in Urban and Rural Scotland, 1861–1901* (Alghero, 2014).

16 D. Armstrong, ‘The Invention of Infant Mortality’, *Sociology of Health & Illness*, 8 (1986), 211–32; Anderton and Leonard, ‘Grammars of Death’; Kunitz, ‘Premises, Premises’.

17 Kunitz, ‘Premises, Premises’.

18 Reid et al., ‘A Century of Deaths’.

19 H. L. Sommerseth, ‘What was Killing Babies in Trondheim? An Investigation of Infant Mortality Using Individual Level Cause of Death Data, 1830–1907’, *Historical Life Course Studies*, 13 (2023), 61–88.

20 For example, the booklet NAS, GRO5/814, Booklet ‘Suggestions as to Death Certification’ (Prepared and Issued by the Registrar-General for Scotland, August 1924, NAS, GRO5/814) provides the preferred nosology and also a list of indefinite or undesirable’ causes which doctors should avoid using.

21 Second Detailed Annual Report of the Registrar-General of Births, Deaths and Marriages in Scotland. BPP 1862 XVIII (2917) xxxiii.

22 Reid et al., ‘A Confession of Ignorance’.

23 In other contexts, however, increasing precision could actually disguise true causes, for example doctors frequently attributed maternal deaths to ‘haemorrhage’ or ‘peritonitis’ without mentioning a recent delivery, while lay-people simply reported that a woman had died in childbirth (Reid and Garrett, ‘Medical Provision’).

24 Alter and Carmichael, ‘Classifying the Dead’, 130.

25 Reid et al., ‘A Confession of Ignorance’, 333.

26 Correspondence re-revised Medical Certificate with Cause of Death and Cause of Stillbirth, NRS, GRO5_1674 p. 244 Item 5.

27 Reid et al., ‘A Confession of Ignorance’, 332; Select Committee on Death Certification, ‘First and Second Report’, xvii.

28 F. Janssen and A. E. Kunst, ‘ICD Coding Changes and Discontinuities in Trends in Cause-Specific Mortality in Six European Countries, 1950–99’, *Bulletin of the World Health Organization*, 82 (2004), 904–13; F. Meslé and J. Vallin, ‘The Effect of ICD-10 on Continuity in Cause-of-Death Statistics. The Example of France’, *Population (english edition)*, 63 (2008), 347; F. Meslé and J. Vallin, ‘Reconstructing Long-Term Series of Causes of Death: The Case of France’, *Historical Methods: A Journal of Quantitative and Interdisciplinary History*, 29 (1996), 72–87; M. Pechholdová et al., ‘Reconstructing Long-Term Coherent Cause-of-Death Series, a Necessary Step for Analyzing Trends’, *European Journal of Population*, 33 (2017), 629–50; M. Pechholdova, ‘Results and Observations from the Reconstruction of Continuous Time Series of Mortality by Cause of Death: Case of West Germany, 1968–1997’, *Demographic Research*, 21 (2009), 535–68; R. H. M. van der Stegen et al., ‘A Novel Time Series Approach to Bridge Coding Changes with a Consistent Solution Across Causes of Death’, *European Journal of Population*, 30 (2014), 317–35; J. Wolleswinkel-van den Bosch et al., ‘Cause-Specific Mortality Trends in The Netherlands, 1875–1992: A Formal Analysis of the Epidemiologic Transition’, *International Journal of Epidemiology*, 26 (1997), 772–81; J. H. Wolleswinkel-Van Den Bosch et al., ‘Reclassifying Causes of Death to Study the Epidemiological Transition in The Netherlands, 1875–1992’, *European Journal of Population/Revue Européenne de Démographie*, 12 (1996), 327–61.

29 J. Bernabeu-Mestre et al., ‘El análisis histórico de la mortalidad por causas: problemas y soluciones’, *Revista de Demografía Histórica*, 21 (2003), 167–93; Revuelta-Eugercios et al., ‘Older Rationales’.

30 Revuelta-Eugercios et al., ‘Older Rationales’.

31 Creating a historic classification system by starting with a modern one is not unique; it is the basis of the widely used historical classification of occupations (HISCO) (M. H. D. van Leeuwen et al., *HISCO: Historical International Standard Classification of Occupations* (Leuven University Press, 2002).

32 WHO | FAQ on ICD, https://web.archive.org/web/20041017011702/http://www.who.int/classifications/help/icdfaq/en/, last accessed 16 December 2023.

33 See https://icd.who.int/browse10/2016/en for the ICD10 coding system in full.

34 Fifth Detailed Annual Report of Registrar General of Births, Deaths and Marriages in Scotland BPP 1864 XVII (3251) xxxv–xxxvii.

35 Fourteenth Annual Report of the Registrar-General (1851) (Registrar General's edition) BPP 1855 98 and 128.

36 It is very unlikely that ‘teething syndrome’ caused a death, but ICD10 not only provides codes and classifications for causes of death, but also for diseases and conditions which might not lead to death.

37 Recent evidence suggests that at least in late nineteenth-century Ipswich, it is unlikely that infants whose death was attributed to teething died from diarrhoea (E. Garrett and A. Reid, ‘What was Killing Babies in Ipswich Between 1872 and 1909?’, *Historical Life Course Studies*, 12 (2022), 173–204.).

38 This gives us potential for 99 additional codes (01, 02 … 98, 99). Numbers above 10 are rarely used, but are included to provide potential for codes for different languages and to give scope for further expansion.

39 If new historic evidence came to light and our interpretation changed, we could allocate the code to a different category in the classification stage.

40 The microscopic diagnosis of tumours became routine at select university hospitals in the UK during the nineteenth century, but there were few specialists involved in the work and only a small fraction of cancer sufferers would have been affected (S. I. Hajdu, ‘A Note from History: Landmarks in History of Cancer, Part 4’, *Cancer*, 118 (2012), 4914–28.). The main advances in cancer detection and diagnosis took place between the 1940s and 1970s (S. I. Hajdu and F. Darvishian, ‘A Note from History: Landmarks in History of Cancer, Part 5’, *Cancer*, 119 (2013), 1450–66; S. I. Hajdu and M. Vadmal, ‘A Note from History: Landmarks in History of Cancer, Part 6’, *Cancer*, 119 (2013), 4058–82.).

41 Determining the Demography of Victorian Scotland Through Record Linkage (ESRC RES-000-23-0128) held at the Cambridge Group for the History of Population and Social Structure, University of Cambridge.

42 P. Gunn and R. Kippen, *Household and Family Formation in Nineteenth-Century Tasmania, dataset of 195 thousand births, 93 thousand deaths and 51 thousand marriages registered in Tasmania, 1838–1899* (Canberra: Australian Data Archive, The Australian National University, 2008).

43 A. Crowther and M. Dupree, *Medical Lives in the Age of Surgical Revolution* (Cambridge: Cambridge University Press, 2007), 256.

44
https://icd.who.int/browse10/2016/en

45 J. Copland, *A Dictionary of Practical Medicine* (London: Longman, Brown, Green, Longmans and Roberts, 1833); R. Dunglison, *Medical Lexicon*, 2nd edn (Philadelphia: Lea and Blanchard, 1839); H. Payne, *A Pocket Vocabulary of Medical Terms (with their Pronounciation), for the use of Registrars, Poor Law Officials*, Etc. (London: Hadden, Best and Co., 1884). We also used Rudy's List of Archaic Medical Terms, available at http://www.antiquusmorbus.com/English/EnglishA.htm.

46 Hardy, ‘Death is the Cure of All Diseases’; Kippen, ‘Incorrect, Loose and Coarse Terms’; B. Luckin, ‘Evaluating the Sanitary Revolution: Typhus and Typhoid in London, 1851–1900’, *Urban Disease and Mortality in Nineteenth Century England* (London: Batsford, 1984), 102–19; A. Tanner, *The Historic Hospital Admission Records Project (HHARP)*, 2013. http://www.hharp.org, last accessed 20 November 2023; Williams, ‘The Reporting and Classification’.

47 WHO, *International Statistical Classification of Diseases and Related Health Problems 10th Revision. Volume 2: Instruction Manual*, 5th edin (Geneva: WHO, 2016).

48 On ACME see: https://www.cdc.gov/nchs/nvss/mmds/about_mmds.htm, last accessed 13 June 2022, although we used a version downloaded in 2014. Another software for resolving multiple codes is IRIS: https://www.bfarm.de/EN/Code-systems/Collaboration-and-projects/Iris-Institute/Iris-software/_node.html, last accessed 13 June 2022.

49 A. Reid et al., ‘Historic Cause of Death Coding and Classification Scheme for Individual-Level Causes of Death - Manual’, 2024; A. Reid et al., ‘Historic Cause of Death Coding and Classification Scheme for Individual-Level Causes of Death - Codes’, 2024; A. Reid et al., ‘Historic Cause of Death Coding and Classification Scheme for Individual-Level Causes of Death – English Language Historic Strings’, 2024.

50 V. Maiolo and A. Reid, ‘Looking for an Explanation for the Excessive Male Mortality in England and Wales Since the End of the 19th Century’, *SSM - Population Health* 11 (2020), 1584; Reid et al., ‘A Confession of Ignorance’; Reid et al., ‘A Century of Deaths’.

51 A. Reid et al., ICD10h Codes.

52 A. Reid et al., ‘Historic Cause of Death Coding and Classification Scheme for Individual-Level Causes of Death – Infant Categorisations’, 2024.

53 The transcription used the tiff image files created over 20 years ago from jacketed fiche images, which had themselves been created from the original records by NRS over 40 years ago. Transcription was contracted to an off-shore company and was set up in a way which flexibly allowed transcribers to report on poor images and unreadable characters (letters). Once the transcription was received, quality assurance checks were performed on a random sample of 1.5 per cent of all records and found a 99.7 per cent accuracy level of the transcription (excluding unreadable characters) over all record sets (births, marriages and deaths). Accuracy was assessed as the percentage of readable (in the opinion of the checker) characters that were correct, as far as the checker could assess. There were two approaches to capturing unreadable characters: where one (or more) letter(s) in a word were unreadable, each of those letters was replaced with @; if the whole word was deemed unreadable, # was inserted. Overall, there were 9,697,656 @ signs recorded (which represents 0.25 per cent of all characters transcribed) and 500,587 # whole words were unreadable across the transcribed record sets.

54 R. Tobin et al., ‘Automatic Coding of Occupation and Cause-of-Death Records’, *International Journal of Population Data Science* (2019).

55 L. V. Perner, *Beyond the Grave*, PhD thesis (Copenhagen: SAXO-instituttet Københavns Universitet, 2023).

56 Reid and Garrett, ‘Medical Provision’.

57 Alter and Carmichael, ‘Classifying the Dead’; L. Bryder, ‘“Not Always One and the Same Thing”: The Registration of Tuberculosis Deaths in Britain, 1900–1950’, *Social History of Medicine*, 9 (1996), 253–65; B. Bynum, ‘Consumption’, *The Lancet* 358 (2001), 676; H. Bynum, *Spitting Blood: The History of Tuberculosis* (Oxford, New York: Oxford University Press, 2015); Hardy, ‘Death is the Cure of All Diseases’.

58 Anderton and Leonard, ‘Grammars of Death’.

59 Garrett and Reid, ‘What was Killing Babies in Ipswich Between 1872 and 1909?’; J. M. Pujadas-Mora, ‘What was Killing Babies in Palma, Spain? Analysing Infant Mortality Patterns Using Individual-Level Cause of Death Data, 1836–1930’, *Historical Life Course Studies* 14 (2024), 82–104; M. Raftakis, ‘What was Killing Babies in Hermoupolis, Greece? An Investigation of Infant Mortality Using Individual Level Causes of Death, 1861–1930’, *Historical Life Course Studies* 12 (2022), 205–32; M. Mühlichen and L. Cilek, ‘What Was Killing Babies in Rostock? An Investigation of Infant Mortality Using Individual-Level Cause-of-Death Data, 1800–1904’, *Historical Life Course Studies* 14 (2024), 16–40; L. Ludvigsen et al., ‘Cause-Specific Infant Mortality in Copenhagen 1861–1911 Explored Using Individual Level Data’, *Historical Life Course Studies* 13 (2023), 9–43; A. Janssens and T. Riswick, ‘What was Killing Babies in Amsterdam? A Study of Infant Mortality Patterns Using Individual-Level Cause of Death Data, 1856–1904’, *Historical Life Course Studies* 13 (2023), 235–64; Sommerseth, ‘What was Killing Babies in Trondheim?’; M. H. Maltesdotter and S. Edvinsson, ‘What was Killing Babies in Sundsvall? A Study of Infant Mortality Patterns Using Individual Level Cause of Death Data, 1860–1892’, *Historical Life Course Studies* 15 (2025), 1–27. A. Reid and A. Janssens, ‘What was Killing Babies in 19th-Century Europe? Categorising Their Deaths Using ICD10h’, *Historical Life Course Studies* 15 (2025), 266–280.

60 R. Woods and N. Shelton, *An Atlas of Victorian Mortality* (Liverpool: Liverpool University Press, 1997).

61 R. I. Woods et al., ‘The Causes of Rapid Infant Mortality Decline in England and Wales, 1861–1921 Part I’, *Population Studies* 42 (1988), 343–66.

62 Anderson considers deaths from typhoid, dysentery, diarrhoea, enteritis and cholera as due to ‘gastro-intestinal diseases’ across the 1856–1901 period. M. Anderson, *Scotland's Populations, from the 1850s to Today* (Oxford: Oxford University Press, 2018), 290.

63 Booklets of Medical Certificates of Cause of Death date 190_ (so meant for use in the 1900s) were provided for doctors (NRS GRO 5/814). These include an insert in red ink entitled ‘Suggestion as to certificates of death from Diarrhoeal Diseases’ and carrying the text: ‘The Royal College of Physicians of Edinburgh has expressed its cordial approval of the recommendation that in Certificates of Death the term “Epidemic Enteritis” should in future be used in place of “Epidemic Diarrhoea”, and its conviction that the general adoption of this recommendation by the Practitioners of Scotland would be highly advantageous in the Interests of Public Health, and of accuracy in the National Records of Mortality’.

64 A. Hardy, *The Epidemic Streets: Infectious Diseases and the Rise of Preventive Medicine 1856–1900* (Oxford University Press, 1993).

65 Alter and Carmichael, ‘Classifying the Dead’, 131.

66
*Ibid*.

## Appendix

Differences between allocation of deaths, 1855–1901, to HistCat categories, comparing published sources (Registrar General) and Digitising Scotland data

**Table:**

	Registrar General (RG)		Digitising Scotland (DS)		Difference		
	Number	% Distribution	Number	% Distribution	Absolute	As % of RG	Absolute difference in % distribution
Childbirth	28,023	0.81	21,857	0.63	6166	22.00	0.18
Circulatory	331,724	9.61	320,700	9.29	11,024	3.32	0.32
Diarrhoea	224,974	6.52	247,822	7.18	−22,848	−10.16	−0.66
Digestive	148,544	4.30	133,754	3.87	14,790	9.96	0.43
Genitourinary	73,457	2.13	67,281	1.95	6176	8.41	0.18
Infectious	409,414	11.86	369,180	10.69	40,234	9.83	1.16
Neoplasms	93,000	2.69	109,420	3.17	−16,420	−17.66	−0.48
Nervous system	243,311	7.05	242,853	7.03	458	0.19	0.01
Old age	246,802	7.15	232,958	6.75	13,844	5.61	0.40
Other	79,398	2.30	102,311	2.96	−22,913	−28.86	−0.66
Perinatal	195,675	5.67	176,295	5.11	19,380	9.90	0.56
Respiratory	632,883	18.33	635,708	18.41	−2825	−0.45	−0.08
Tuberculosis	463,675	13.43	445,068	12.89	18,607	4.01	0.54
External	118,257	3.42	92,296	2.67	25,961	21.95	0.75
NA/ill-defined	163,626	4.74	254,842	7.38	−91,216	−55.75	−2.64
Total	3,452,763	100.00	3,452,345	100.00	418	0.01	100.00

Sources: Digitising Scotland data; R.J. Davenport. ‘Annual deaths by cause, age, and sex in Scotland, 1855–1949’, https://www.campop.geog.cam.ac.uk/research/projects/longrundeathcauses/ (2012).

